# Enhanced Extraction Efficiency of Flavonoids from *Pyrus ussuriensis* Leaves with Deep Eutectic Solvents

**DOI:** 10.3390/molecules27092798

**Published:** 2022-04-28

**Authors:** Jong Woo Lee, Hye Yoon Park, Junseong Park

**Affiliations:** 1 Department of Engineering Chemistry, Chungbuk National University, Chungbuk, Cheongju 28644, Korea; qoohji@naver.com; 2 Biological and Genetic Resources Assessment Division, National Institute of Biological Resources, Incheon 22689, Korea; rejoice077@korea.kr

**Keywords:** *Pyrus ussuriensis* leaves, flavonoids, deep eutectic solvent, response surface methodology, green extraction

## Abstract

In this study, deep eutectic solvents (DESs) were synthesized using different ratios of choline chloride (CC) and dicarboxylic acids, and their eutectic temperatures were determined. The DES synthesized using CC and glutaric acid (GA), which showed a higher extraction efficiency than conventional solvents, was used for the extraction of flavonoid components from *Pyrus ussuriensis* leaves (PUL), and the extraction efficiency was evaluated using the response surface methodology. The flavonoid components rutin, hyperoside, and isoquercitrin were identified through high-performance liquid chromatography (HPLC), equipped with a Waters 2996 PDA detector, and HPLC mass spectrometry (LC-MS/MS) analyses. The optimum extraction was achieved at a temperature of 30 °C using DES in a concentration of 30.85 wt.% at a stirring speed of 1113 rpm and an extraction time of 1 h. The corresponding flavonoid content was 217.56 μg/mL. The results were verified by performing three reproducibility experiments, and a high significance, with a confidence range of 95%, was achieved. In addition, the PUL extracts exhibited appreciable antioxidant activity. The results showed that the extraction process using the DES based on CC and GA in a 1:1 molar ratio could effectively improve the yield of flavonoids from PUL.

## 1. Introduction

Eco-friendly chemistry has recently attracted considerable attention, and “green solvents” based on natural substances are being proposed as useful alternatives to conventional organic solvents [1]. Among them, deep eutectic solvents (DESs) are highly suitable for the extraction of a wide range of natural compounds [2]. Natural DESs (NADESs) occur in a variety of living cells and play an important role as an alternative medium for the biosynthesis, transport, and storage of natural products [3]. DESs are typically composed of nontoxic substances occurring naturally in some plants. These solvents can be used to extract naturally occurring compounds that can be incorporated directly into food formulations without performing additional separation steps. Therefore, DESs have significant advantages over conventional solvents. In addition, several DESs have been found to increase the stability of natural compounds during extraction and storage [4]. Therefore, studies have been performed to determine the potential utility of DESs as extraction media for sparingly soluble bioactive compounds that are not amenable to aqueous extraction [5,6]. DESs consist of mixtures of a wide range of hydrogen bond donor (HBD) and hydrogen bond acceptor (HBA) species. Typical HBAs are nontoxic quaternary ammonium salts or amino acids, such as alanine, proline, and glycine, and HBDs are primarily organic acids, such as oxalic, lactic, and malic acids, or carbohydrate-based substances, such as glucose, fructose, and maltose. Alcohol, amine, aldehyde, ketone, and carboxylate functionalities are highly versatile and can be used to prepare custom solvents of virtually unlimited combinations, as they are capable of both HBA and HBD functions. The nature of the interaction that occurs depends on the type of solid material that forms the liquid [7,8,9]. The temperature, viscosity, stability, and solvent melting properties are related to the strength of the hydrogen bonds [10].

DESs are typically used to extract hydrophobic components that cannot be efficiently extracted with water, as well as to extract hydrophilic components [11,12]. Flavonoids are important active compounds that exist in more than 5000 natural states, and are known to have excellent antioxidant properties because they effectively eliminate active oxygen species [13,14,15]. Recently, as the role of active oxygen species in the progression of degenerative diseases has been gradually recognized, research has been underway on the development of antioxidants that can arrest and eliminate active oxygen species [16,17,18].

*Pyrus bretschneideri* (genus: *Pyrus*, family: *Rosaceae*), the third most important temperate climate fruit species after grapes and apples, has been cultivated in more than 50 countries since it was first cultivated in China 2000 years ago [19,20]. The genus *Pyrus* contains twenty-two widely recognized primary species, including at least six wild species and three interspecific hybrids [21]. Pear trees are a deciduous tree plant species belonging to the genus *Pyrus,* and are one of the representative cultivated varieties that have been introduced and grown nationwide in Korea [22]. *Pyrus ussuriensis* is a deciduous tree belonging to the family *Rosaceae*. Parts of this tree have been used in the private sector as medicinal products to treat vomiting, diarrhea, and fever. The fruits have been historically enjoyed by our ancestors [23]. PUL are used as a traditional herbal medicine for treating asthma, coughs, and fever, and although they are effective in treating atopic dermatitis, reports on the efficacy of certain components are insufficient [24]. The separation of isoprenoid alcohols and polyphenols from the leaves of *Pyrus ussuriensis* has been reported, along with the separation of flavonoids from the stems. However, more studies have been focused on the well-known Korean tree *Pyrifolia Nakai* [25,26].

The main purpose of this study is to synthesize DESs using choline chloride (CC) and dicarboxylic acids, and apply them to extracting flavonoids from PUL. In the process of extracting flavonoid components from PUL, the conditions that can maximize the extraction efficiency have been determined using the response surface methodology (RSM).

## 2. Results and Discussion

### 2.1. Chemical Profiling of PUL Extracts

**Figure 1:**
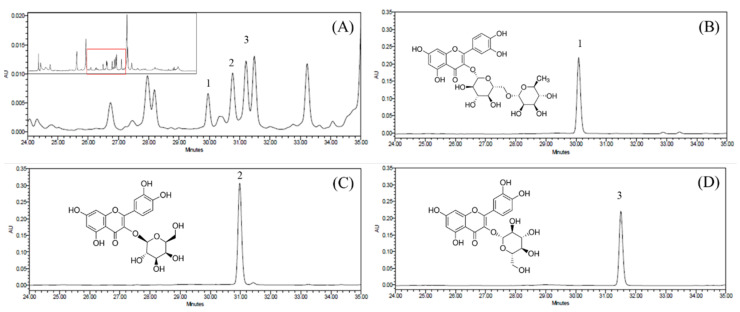
HPLC chromatograms of PUL extract and standard samples. Detection interval of (**A**) extract, (**B**) rutin, (**C**) hyperoside, and (**D**) isoquercitrin.

A total of 30.02 g of dried PUL was extracted to obtain 4.24 g of freeze-dried extract powder (yield: 14.12%). To analyze the active compounds present in the PUL, the extract was subjected to liquid chromatography–tandem mass spectrometry (LC-MS/MS) analysis. On performing LC-MS/MS analysis, valid peaks corresponding to the flavonoid components were detected in the range of 35–37 min at a wavelength of 270 nm. As can be observed in Figure S1A, the *m*/*z* value of [M-H]^-^ was 609.15 at 35.0 min. The *m*/*z* value of [M-H]^−^ was 463.09 at 35.2 min and 35.6 min (Figure S1B,C). From the results of the MS analysis, rutin, hyperoside, and isoquercitrin were determined to be the flavonoid components of PUL. In this study, the HPLC-MS/MS results show that rutin, hyperoside, and isoquercitrin are the active flavonoid components present in the PUL extracts. To confirm the presence of the flavonoids in the PUL extracts, standard samples of rutin, hyperoside, and isoquercitrin were subjected to HPLC analysis equipped with a Waters 2996 PDA detector, and the resulting chromatograms were compared with that obtained for the PUL extract (Figure 1). The retention times of the standard rutin, hyperoside, and isoquercitrin samples are consistent with those of the peaks 1, 2, and 3 of the PUL extract.

### 2.2. Preparation of DES and Selection of DES by Extraction Efficiency

**Figure 2:**
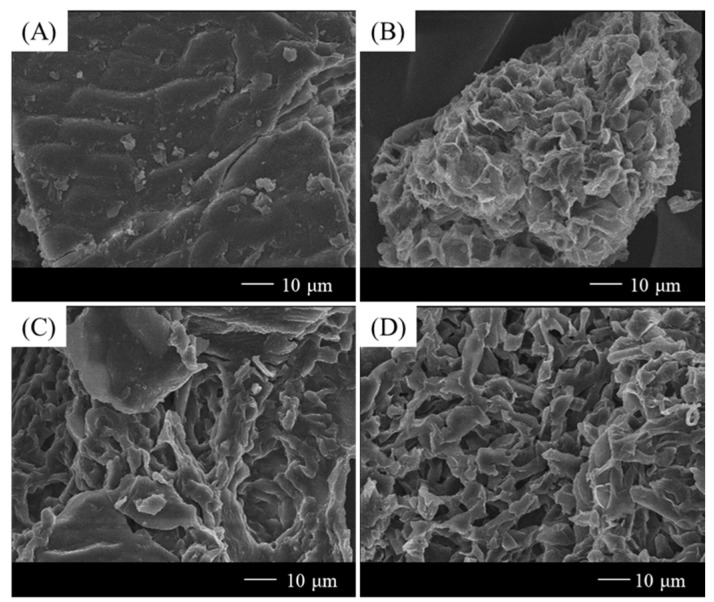
Configuration of PUL surface after extraction using different solvents. (**A**) PUL surface before extraction; (**B**) PUL surface after extraction with water; (**C**) PUL surface after extraction with DES synthesized using CC and malonic acid; (**D**) PUL surface after extraction with DES synthesized using CC and GA.

Five types of DESs were synthesized, using CC as the HBA. Different dicarboxylic acids were employed as the HBDs. All the HBDs contain two carboxyl groups, but differ in the total number of carbon atoms. The efficiencies of the solvent extractions of the flavonoids from PUL, using the various DESs, are listed in Table 1. The extraction efficiency of the flavonoids is expressed as the sum of the extraction contents of three components: rutin, hyperoside, and isoquercitrin. Three standard samples of each extract were evaluated using HPLC with a correlation coefficient of 0.99. The contents of the flavonoids in the extract were determined using this standard curve. All the extraction efficiencies with the DESs were higher than those obtained with water (used for comparison), except for that pertaining to the DES synthesized using adipic acid. The extraction efficiencies of PUL with the malonic- and glutaric acid (GA)-based DESs were significantly higher than those with the other DESs. Therefore, the DES prepared using GA and CC was selected for the extraction of PUL prior to the optimization of the extraction condition. The HBDs and HBAs in the DES can interact with the cellulose and lignin in the plant cell walls to promote loosening of the cell wall structure and enable easy extraction of the intracellular components. A higher maximum extraction efficiency was achieved with the DES than without it, which was consistent with previous reports [27]. In addition, the current results show that when CC is used as the HBA, the extraction efficiency varies with the types of HBD used. Higher extraction efficiencies were achieved using DESs based on structurally similar dicarboxylic acids with odd numbers of carbon atoms. It has been confirmed that the structures of the HBDs affect the extraction efficiency, and it can be observed in Table 1 that the molar ratios vary depending on the structures. The current results also indicate that the eutectic temperature is determined by the structure of the HBD. Field emission scanning electron microscopy (FE-SEM) studies indicated the penetration of trace amounts of the solvents on the PUL surface during the extraction process. The FE-SEM images of the pre- and post-extraction PUL samples are shown in Figure 2. As can be observed in Figure 2A, there was no solvent penetration on the surface of the pre-extraction PUL, while traces of solvent penetration were observed when the extraction was performed with water (Figure 2B). The FE-SEM images of the PUL surfaces after DES extraction (Figure 2C,D) indicate the occurrence of sufficient solvent penetration. The high solubility of the plant cell wall components, such as lignin, cellulose, and flavonoids, in the DES facilitated the penetration of the solvent, which led to structural changes in the overall leaf surface. The efficient penetration of plant tissues by the DES prepared using GA and CC proves that it can serve as an environmentally friendly solvent for isolating natural active components. Based on the penetration, it can be concluded that the synthesized covalent solvent is sufficiently functional.

### 2.3. Optimization of Extraction Conditions for Flavonoids by RSM

Four factors were considered for optimizing the extraction conditions of the DES prepared using GA and CC: temperature, extraction time, DES content, and stirring speed. According to the results of the single-factor experiment (Figure S2), the four factors were subjected to the Box–Behnken design (BBD) analysis. The three levels of extraction temperature were 30, 60, and 90 °C, while those of the extraction time were 1, 24.5, and 48 h. The three levels of DES content were 10, 50, and 90%, and those of the stirring speed were 450, 850, and 1250 rpm. The design parameters were determined using the Design-Expert software and the results are listed in Table 2. The Design-Expert software was used to simulate the results of the BBD analysis. The obtained quadratic polynomial regression equation for the extraction efficiency Y, as the sum of the contents of rutin, hyperoside, and isoquercitrin, versus the temperature (A), extraction time (B), DES content (C), and stirring speed (D) was as follows:

*Y (content of flavonoids)* = 147.43 − 16.44*A* − 10.20*B* − 28.63*C* + 25.72*D* − 32.66*AB* + 1.18*AC* − 15.59*AD* + 10.62*BC* − 0.6583*BD* −9.24*CD* + 20.68*A*^2^ + 10.77*B*^2^ − 48.72*C*^2^ + 13.77*D*^2^

**Figure 3:**
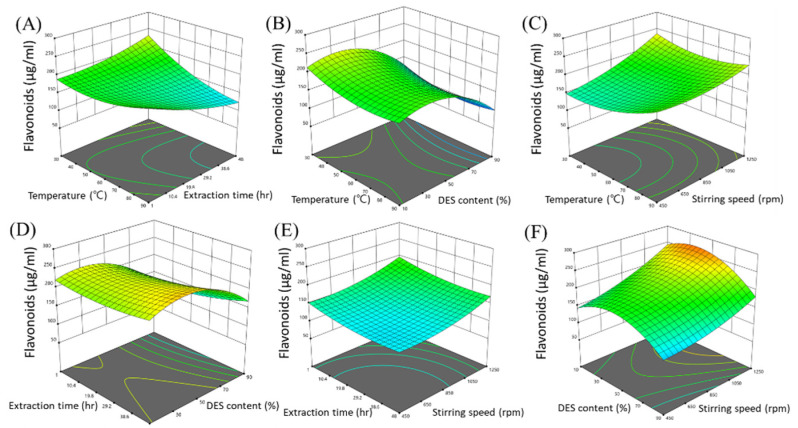
Response surface in Box–Behnken experimental design for the four factors influencing the extraction of flavonoids. (**A**) Effect of temperature and extraction time; (**B**) effect of temperature and DES content; (**C**) effect of temperature and stirring speed; (**D**) effect of extraction time and DES content; (**E**) effect of extraction time and stirring speed; and (**F**) effect of DES content and stirring speed.

The results of the variance analysis of the above equation are listed in Table 3. According to the analysis of variance (ANOVA), the *p*-value (0.0441) of the four-factor global model was significant. The model F-value of 2.57 implied that the model was significant. The quadratic coefficients (A^2^, B^2^, C^2^, and D^2^) also had a significant effect, while the linear coefficients (A, B, C, and D) and terms representing the interaction between two factors had a negligible effect. Therefore, the above model can be employed to predict the extraction efficiency of the DES toward PUL. In the DES extraction, the content of DES was found to be the variable with the highest influence on the content of flavonoids. It also affected the order of the stirring speed, extraction temperature, and extraction time. The results confirmed that all the interactions, except that between the extraction temperature and extraction time, were insignificant. Figure 3 depicts a three-dimensional (3D) response surface curve based on the regression polynomial. It represents the range of three independent variables, while the fourth variable is held at level 0. As can be observed in Figure 3A, the extraction efficiency increased as the extraction temperature and time increased. On the other hand, it tended to increase initially and then decrease, depending on the DES content. The maximum extraction efficiency was achieved at a DES content of 30.85%. At low stirring speeds, the extraction efficiency was found to increase with temperature. However, when the stirring speed is fast, the extraction efficiency decreases as the temperature increases, and then increases again when the temperature reaches 60 °C. The optimal extraction conditions predicted from the three-dimensional response surface curves were as follows: extraction temperature: 30°C, extraction time: 1 h, DES content: 30.85%, and stirring speed: 1113.1 rpm. The optimized conditions corresponded to a predicted flavonoid content of 217.515 μg/mL. Three confirmatory tests, performed under the predicted conditions, showed high significance, with error rates within 5%, and flavonoid yields of 223.37, 219.10, and 227.11 μg/mL.

### 2.4. Evaluation of Antioxidant Activity

**Figure 4:**
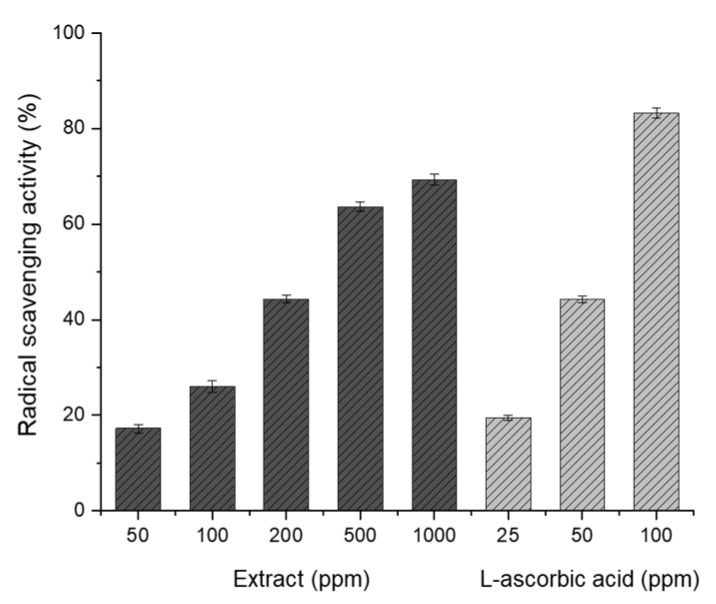
DPPH radical scavenging activity of *PUL* extract under optimal extraction conditions.

The antioxidant activity of the flavonoids extracted from the PUL under the optimum conditions was evaluated at various concentrations. The flavonoid concentration corresponding to 50% free radical scavenging activity was calculated based on L-ascorbic acid, which constituted the positive control group. As can be observed in Figure 4, the 2,2-diphenyl-1-picrylhydrazyl (DPPH) free radical scavenging activity increases as the concentration of the extract is increased. However, it does not increase when the maximum concentration is reached. The concentrations of L-ascorbic acid (positive group) and the eluted extract corresponding to 50% DPPH radical scavenging activity were 59.54 and 299.68 ppm, respectively. The antioxidant activity of the active component of the PUL extracted using the DES was approximately 1/6 that of L-ascorbic acid. It was, thus, confirmed that the active components of the PUL extracted using the DES had potential utility as antioxidants.

### 2.5. Evaluation of Anti-Inflammatory Effectiveness

**Figure 5:**
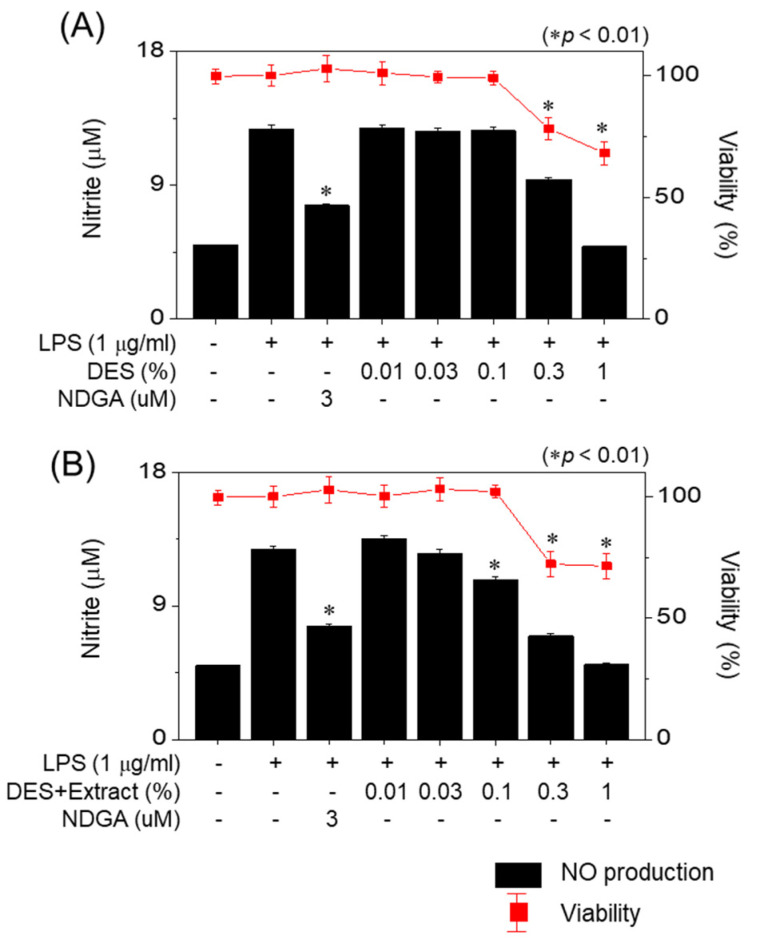
Effect of DES and PUL extract on the production of NO in LPS-stimulated RAW 264.7 cells. (**A**) DES synthesized using CC and GA; (**B**) PUL extract obtained using DES synthesized from CC and GA.

Inflammatory reactions are defense mechanisms against external invaders, which produce a variety of inflammatory factors. Among them, nitric oxide (NO) is a highly reactive biomolecule. When cells are stimulated by lipopolysaccharides (LPSs), they produce NO in the presence of inducible nitric oxide synthase (iNOS) [28,29]. The inhibition of NO production in RAW 264.7 cells stimulated by LPSs is illustrated in Figure 5. Both the DES and PUL extract obtained using the DES showed cytotoxicity at concentrations exceeding 0.3%. As can be observed in Figure 5A, the DES did not inhibit the generation of NO at a concentration of 0.1%, while the PUL extracts obtained using the DES significantly inhibited NO generation (up to 26.1%) at the same concentration (Figure 5B). The anti-inflammatory properties of the active components of the PUL extracted using the DES were, thus, demonstrated.

## 3. Materials and Methods

### 3.1. Materials

Dried PUL were provided by the National Institute of Biological Resources. Choline chloride (≥99.0%), oxalic acid, anhydrous (≥98.0%), and malonic acid (≥99.0%) were supplied by Samchun Chemical Co., Seoul, Korea. Succinic acid (≥99.5%) was purchased from Junsei Chemical Co., Japan, Glutaric acid (≥99.0%) was supplied by Daejung Chemicals and Metals, Gyunggi-do, Korea. Adipic acid (≥99.0%) was procured from Sigma Aldrich, USA. Acetonitrile (ACN, CH_3_CN, 99.9%, Fisher Chemical, Loughborough, UK) was used as a solvent for the mobile phase of HPLC, and acetic acid (CH_3_COOH, ACS reagent, Sigma Aldrich, St. Louis, MO, USA) was used as an additive.

### 3.2. Extraction and Determination of Flavonoids in PUL

PUL were dried and crushed up to 50 mesh. The crushed leaves (30 g) were extracted by stirring for 2 h at 30 °C with 600 mL of 70% ethanol, followed by filtration. The extract was obtained in powder form by concentrating and freeze-drying. The extract was subjected to HPLC analysis on a Waters 2695 separation module equipped with a Mightysil RP-18GP column (KANTO CHEMICAL, Japan). The sample was examined at a wavelength of 270 nm (UV) using a detector (Tunable Absorbance Detector, Waters, USA). Two solution systems were used in the HPLC mobile phase: the A solution (acetonitrile containing 0.1 wt.% acetic acid) and B solution (distilled water containing 0.1 wt.% acetic acid). The analysis was performed at a flow rate of 1.0 mL/min. The mobile phase B was eluted from 0 min to 100% and to be 70% to 40 min, 40% from 40 to 45 min, 10% to 50 min, and 100% to 60 min.

### 3.3. Preparation of DES

CC, a naturally occurring quaternary ammonium salt, was used as the HBA, and a dicarboxylic acid with two carboxy groups was used as the HBD to prepare the DES. The dicarboxylic acids were classified according to their carbon numbers, and the ratios were determined experimentally from oxalic (C2) to adipic acid (C6). The corresponding DESs were synthesized and used for extraction. In the ratio determination experiment, a thermomixer (Eppendorf thermomixer comfort, Eppendorf, Germany) was used to mix the HBA and HBD at ratios ranging from 3:1 to 1:3 at room temperature. The HBA and HBD were allowed to react under stirring at 950 rpm for 30 min. The temperature was raised by 2 °C/30 min until the formation of a transparent liquid, and the process was recorded. After determining the ratio, the mixture was heated at 80 °C for 2 h to obtain a uniform and stable transparent solution, which was aged in an oven for 24 h at 60 °C prior to use in extraction. The characteristic properties of the synthesized DESs are listed in Table 1. The molar ratio of the HBA and HBD used to synthesize each DES is included in Table 1.

### 3.4. Extraction with DES

The extractions with the DESs and purified water as extraction solvents were performed as follows: 30 mg of powdered PUL extract was mixed with the solvent and stirred at 40 °C at 950 rpm for 60 min. Each extract was subsequently centrifuged at 13,000 rpm to separate the supernatant and filtered through a syringe filter prior to analysis. The extract was subjected to HPLC analysis to determine the content of flavonoids.

### 3.5. Optimization of the Extraction Condition using the Box–Behnken Design

Based on the results of the screening test, the BBD experimental design of the RSM was performed using the Design-Expert 12.0 software to optimize the four selected factors, extraction temperature (°C), extraction time (h), DES content (%), and stirring speed (rpm), that influenced the extraction of PUL and could enhance the extraction efficiency of the flavonoids. The four independent factors were investigated at three different levels (−1, 0, and +1). The complete experimental design consisted of 29 runs. Each run was repeated three times.

### 3.6. SEM Analysis

The native PUL samples (prior to extraction) and those subjected to extraction with water, the DES synthesized from CC and malonic acid, and the DES synthesized from CC and GA were dried at 60 °C for 48 h. The surface characteristics of the samples were observed through a field emission scanning electron microscope (ULTRAPLUS, Carl Zeiss NTS GmbH, Oberkochen, Germany). The effects of different extraction solvents on the surface structure were analyzed.

### 3.7. Antioxidant Activity

The antioxidant activity of the PUL extracts obtained using the DESs synthesized from CC and different dicarboxylic acids was evaluated using the DPPH assay. DPPH is a stable radical that exists as a purple solution, which turns bile yellow as the free radicals are eliminated by hydrogen or electron donors. The radical scavenging activity was estimated by measuring the change in absorbance [30,31]. L-ascorbic acid was used as a positive control to compare the scavenging activity. DPPH was dissolved in ethanol at a concentration of 100 μM. L-ascorbic acid solutions of 50, 100, and 200 ppm concentrations were prepared, and the samples to be tested were diluted to the desired concentrations. Each test solution (10 μL) was mixed with the same amount of ethanol in a 96-well plate. To each of these solutions, 190 μL of DPPH solution (100 μM) was added. After 30 min of reaction at room temperature, the absorbance was measured at 530 nm using a microplate reader. The DPPH free radical scavenging activity was calculated according to the following equation:

$$AA\%=100-\left[\frac{\left(Abs_{sample}-Abs_{blank}\right)\times 100}{Abs_{control}}\right]$$

*Abs_sample_* is the absorbance of the sample in which the extract and the DPPH solution are mixed, *Abs_blank_* is the absorbance inherent in the extract, and *Abs_control_* is the absorbance of the DPPH solution.

### 3.8. Anti-Inflammatory Effectiveness

RAW 264.7 cells were loaded on a 96-well plate at a concentration of 1 × 105 cells/well and incubated for 1 d in a 37 °C incubator. Lipopolysaccharide (LPS) was added at a concentration of 500 ng/mL to induce NO production, and the samples were allowed to react for 24 h. After the reaction, the upper solution layer was reacted with Griess reagent, and the absorbance was measured at 540 nm to estimate the NO production. The cells were treated with 5 mg/mL of 3-(4,5-dimethylthiazol-2-yl)-2,5-diphenyl tetrazolium bromide (MTT) reagent and incubated for an additional hour. The cytotoxicity was determined by removing all the upper fluid and treating it with 100 μL DMSO to completely dissolve the cells. Subsequently, the absorbance was measured at 550 nm. In addition, the significance of the test substance was determined by the two-sample test to meet the biological statistical criterion of (α): 1%. It treated nordihydroguaiaretic acid (NDGA) as a benign control group [28,29].

## 4. Conclusions

In this study, the flavonoid contents of PUL were investigated, and the PUL were discovered to be rich in flavonoids, such as rutin, hyperoside, and isoquercitrin. Extraction using different DESs based on CC and dicarboxylic acids was found to effectively improve the extraction efficiency of the three flavonoids from the PUL. Among the different DESs tested, the DES based on CC and GA exhibited the highest extraction efficiency. The extraction conditions were optimized using the BBD analysis. According to these conditions, the highest extraction efficiency (217.515 μg/mL) could be achieved by performing the extraction of PUL for 1 h at a temperature of 30 °C and stirring speed of 1113.1 rpm, using the DES based on CC and GA at a concentration of 30.85 wt.%. The extraction efficiency achieved using the DES was more than twice as high as that of aqueous extraction. In addition, the PUL extract obtained using the DES synthesized from CC and GA exhibited appreciable antioxidant activity, which was approximately 1/6 that of L-ascorbic acid. The potential utility of the PUL extracts as antioxidants was, thus, demonstrated. The PUL extract obtained using the DES synthesized from CC and GA significantly inhibited NO generation (up to 26.1%) at a concentration of 0.1%. These results indicated the anti-inflammatory properties of the active components of the PUL extracted using the DES.

## Figures and Tables

**Table 1:** Extraction efficiency of flavonoids from *PUL*.

HBD, Carbon Number	Choline Chloride/HBD (Molar Ratio)	Melting Point (°C)	Flavonoid Contents * (μg/mL)
Control (H_2_O)	Control	-	105.926 ± 1.652
Oxalic acid (C_2_)	2: 1	75	150.586 ± 3.072
Malonic acid (C_3_)	1: 1	69	162.169 ± 3.016
Succinic acid (C_4_)	2: 1	80	155.945 ± 2.070
Glutaric acid (C_5_)	1: 1	60	171.326 ± 3.615
Adipic acid (C_6_)	2: 1	84	32.113 ± 0.353

* Flavonoid contents are the sum of contents of the three components (rutin, hyperoside, and isoquercitrin).

**Table 2:** Design and experimental response values based on BBD analysis.

Run	Factor				Flavonoid Content (μg/mL)
	Temperature (A, °C)	Extraction Time (B, h)	DES Content (C, %)	Stirring Speed (D, rpm)	
1	30	24.5	50	450	126.1
2	30	24.5	10	850	118.6
3	60	1	90	850	79.0
4	60	24.5	90	450	93.4
5	90	24.5	50	1250	223.0
6	90	24.5	10	850	95.8
7	60	24.5	50	850	149.6
8	60	48	50	450	147.2
9	60	24.5	90	1250	83.9
10	60	24.5	50	850	139.9
11	60	24.5	50	850	147.4
12	90	24.5	90	850	76.4
13	90	48	50	850	114.8
14	30	24.5	90	850	94.5
15	60	48	50	1250	157.3
16	30	48	50	850	221.3
17	30	24.5	50	1250	291.1
18	60	1	10	850	173.1
19	60	1	50	1250	151.9
20	60	48	10	850	135.2
21	60	24.5	50	850	147.6
22	60	24.5	50	850	152.6
23	60	48	90	850	83.6
24	90	1	50	850	231.3
25	30	1	50	850	207.2
26	90	24.5	50	450	120.3
27	60	1	50	450	139.2
28	60	24.5	10	450	152.1
29	60	24.5	10	1250	179.7

**Table 3:** ANOVA statistical results.

Source	Sum of Squares	Degree of Freedom	Mean of Square	F-Value	*p*-Value
model	53,142.77	14	3795.912143	2.57	0.0441
A-A	3241.96	1	3241.96	2.2	0.1606
B-B	1248.81	1	1248.81	0.8457	0.3733
C-C	9834.27	1	9834.27	6.66	0.0218
D-D	7936.66	1	7936.66	5.38	0.0361
AB	4266.84	1	4266.84	2.89	0.1112
AC	5.53	1	5.53	0.0037	0.9521
AD	972.46	1	972.46	0.6586	0.4306
BC	451.55	1	451.55	0.3058	0.589
BD	1.73	1	1.73	0.0012	0.9732
CD	341.66	1	341.66	0.2314	0.6379
A^2^	2775.34	1	2775.34	1.88	0.192
B^2^	751.98	1	751.98	0.5093	0.4872
C^2^	15,394.76	1	15,394.76	10.43	0.0061
D^2^	1229.1	1	1229.1	0.8324	0.377
Residual	20,672.24	14	1476.588571		
Lack of Fit	20,584.39	10	2058.439	93.73	0.0003
Pure Error	87.85	4	21.9625		
Cor. Total	73,815.01	28			

## Data Availability

Not applicable.

## Supplementary Materials

The following supporting information can be downloaded at: https://www.mdpi.com/article/10.3390/molecules27092798/s1: Figure S1: liquid chromatogram of PUL extract and mass spectra of flavonoid peaks; Figure S2: effect of extraction conditions on extraction efficiency of DES.
