# Supplementary material for: Enhanced Extraction Efficiency of Flavonoids from Pyrus ussuriensis Leaves with Deep Eutectic Solvents
Source: Molecules. 2022 Apr 28;27(9):2798. doi: 10.3390/molecules27092798 (PMC9103983; doi:10.3390/molecules27092798)
Supplement: Supplementary file 1 [file molecules-27-02798-s001.zip › molecules-1641077-supplementary.pdf]

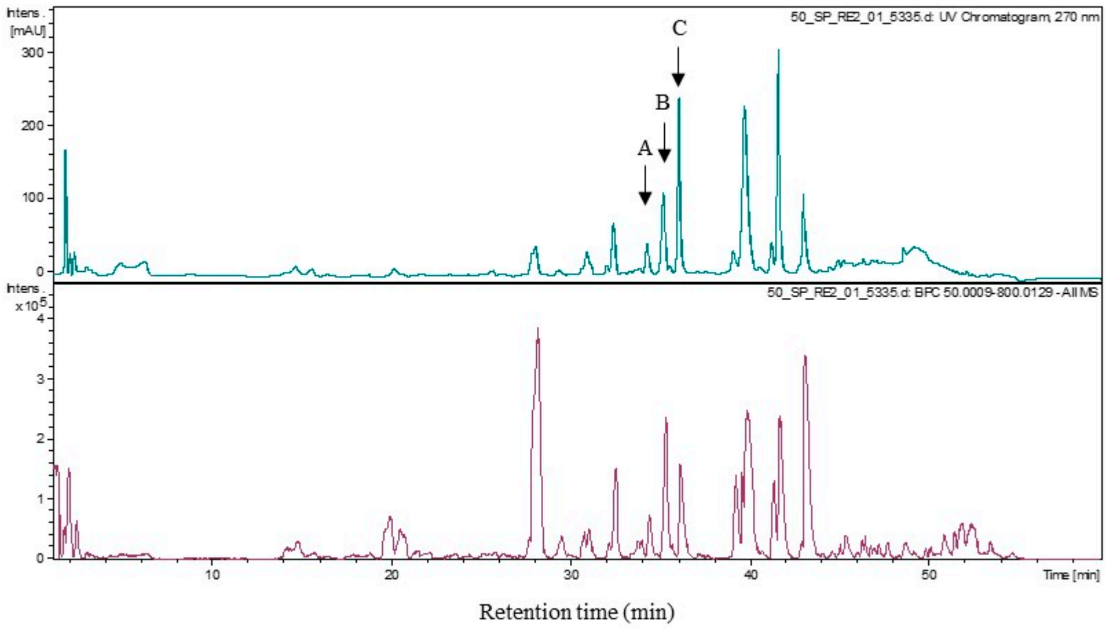

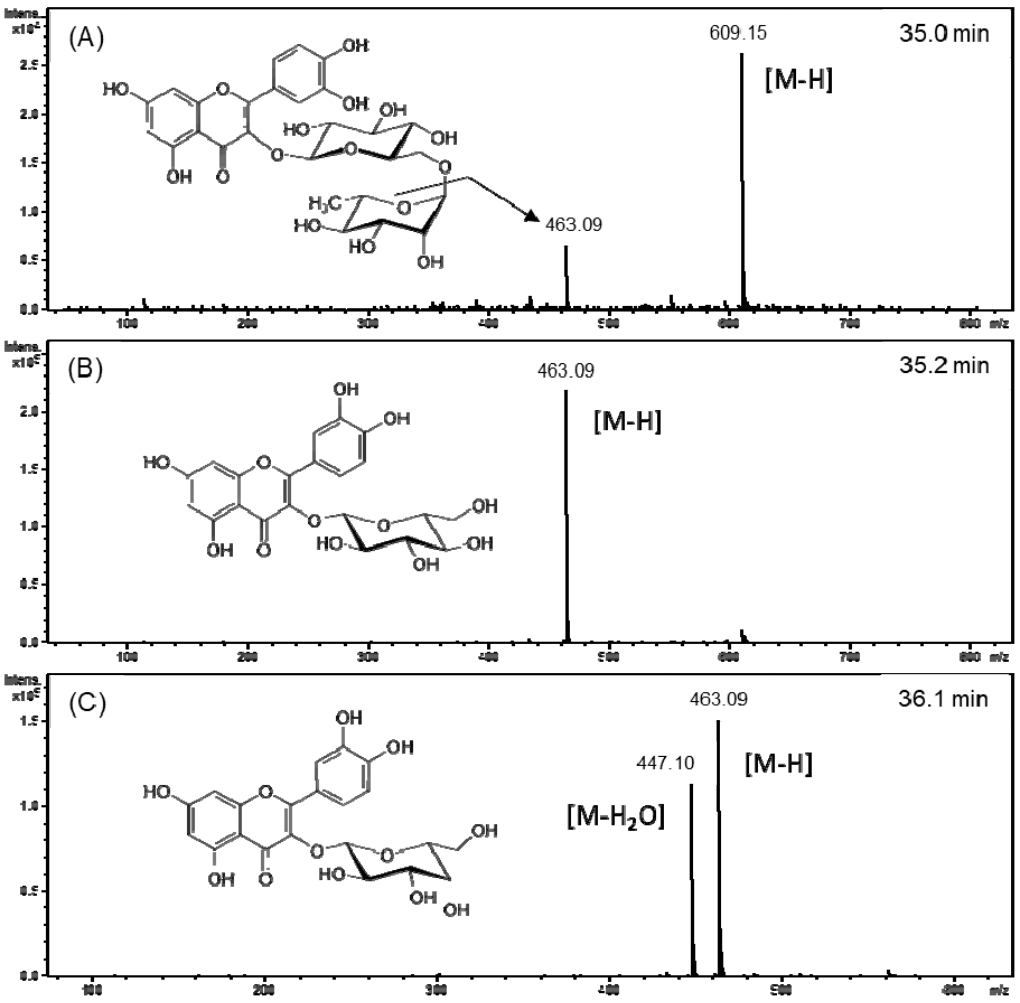

Figure S1. Liquid chromatogram of the PUL extract and mass spectra with the flavonoid peak of (A) rutin, (B) hyperoside and (C) isoquercitrin in the liquid chromatogram.

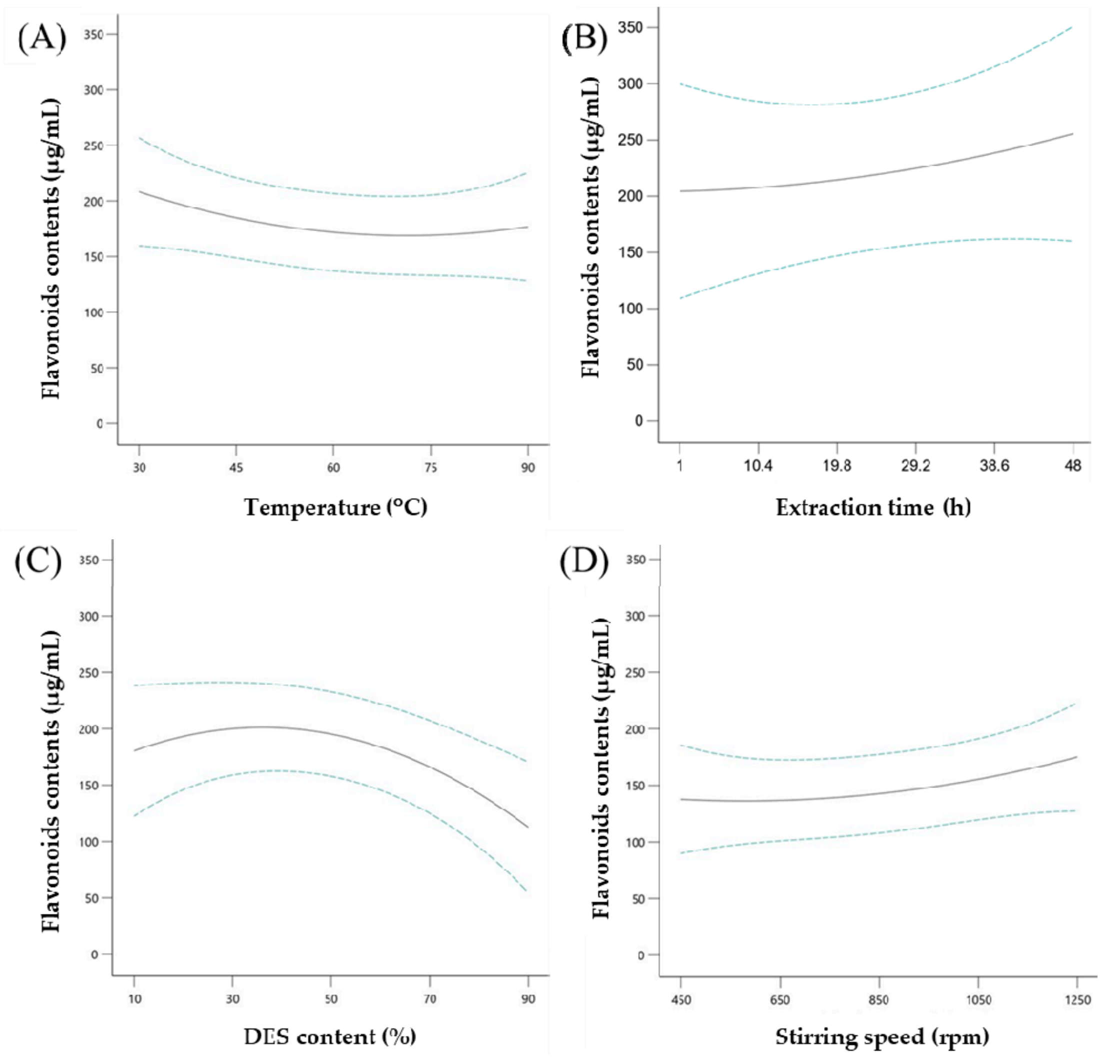

Figure S2. Effects of flavonoid contents on extraction conditions of the DES prepared using GA and CC in terms of (A) extraction temperature; (B) extraction time; (C) DES content; (D) stirring speed.

*Flavonoid contents is the sum of contents of the three components (rutin, hyperoside, and isoquercitrin)
